# A Novel Algorithm for Detecting Protein Complexes with the Breadth First Search

**DOI:** 10.1155/2014/354539

**Published:** 2014-04-10

**Authors:** Xiwei Tang, Jianxin Wang, Min Li, Yiming He, Yi Pan

**Affiliations:** ^1^School of Information Science and Engineering, Central South University, Changsha 410083, China; ^2^School of Information Science and Engineering, Hunan First Normal University, Changsha 410205, China; ^3^Department of Computer Science, Georgia State University, Atlanta, GA 30302-4110, USA

## Abstract

Most biological processes are carried out by protein complexes. A substantial number of false positives of the protein-protein interaction (PPI) data can compromise the utility of the datasets for complexes reconstruction. In order to reduce the impact of such discrepancies, a number of data integration and affinity scoring schemes have been devised. The methods encode the reliabilities (confidence) of physical interactions between pairs of proteins. The challenge now is to identify novel and meaningful protein complexes from the weighted PPI network. To address this problem, a novel protein complex mining algorithm ClusterBFS (Cluster with Breadth-First Search) is proposed. Based on the weighted density, ClusterBFS detects protein complexes of the weighted network by the breadth first search algorithm, which originates from a given seed protein used as starting-point. The experimental results show that ClusterBFS performs significantly better than the other computational approaches in terms of the identification of protein complexes.

## 1. Introduction

Protein complexes are molecular aggregations of proteins assembled by multiple protein-protein interactions. Many proteins are functional only after they are assembled into a protein complex and interact with other proteins in this complex [1–4]. The vast amount of genes and proteins that participate in biological networks imposes the need for determination of protein complexes within the network in order to reduce the complexity, while these complexes will be the first step in deciphering the composite genetic or cellular interactions of the overall network.

High-throughput experimental technologies, along with computational predictions, have produced a large amount of protein interactions [5–11], which make it possible to uncover protein complexes from protein-protein interaction (PPI) networks. Pair-wise protein interactions can be modeled as a graph or network, where vertices represent proteins and edges are protein-protein interactions. Protein complexes are groups of proteins that interact with one another, so they generally correspond to dense subgraphs in PPI networks. Different research groups have developed a wealth of algorithms to identify protein complexes from the PPI networks [12–18]. In these approaches, the protein networks are considered as unweighted graphs. These methods work well on PPI networks and extract successfully protein complexes. Nevertheless, it has been noticed that protein interaction data produced by high-throughput experiments are often associated with high false positive rate and false negative rate due to the limitations of the associated experimental techniques, which may have a negative impact on the complex discovery algorithms [19–23].

In order to address that particular question, a number of data integration and affinity scoring schemes have been devised [10, 23–30]. In the paper of Gavin et al. [11], the weights of the interactions were defined by using the so-called socio-affinity index introduced in [11] that is based on the log-odds of the number of times two proteins were observed together in a purification, relative to the expected frequency of such a cooccurrence based on the number of times the proteins appeared in purifications. Krogan et al. [10] have used MALDI-TOF mass spectrometry and LC-MS/MS to identify protein-protein interactions, based on the observation that either mass spectrometry method often fails to identify a protein, and the usage of two independent methods can increase the coverage and confidence of the obtained interactome. The results of the two methods were combined by supervised machine learning methods with two rounds of learning, using hand-curated protein complexes in the MIPS reference database as a gold standard dataset. Collins et al. [23] have combined the experimentally derived PPI networks of Krogan et al. [10] and Gavin et al. [11] by re-analyzing the raw primary affinity purification data of these experiments using a novel scoring technique called purification enrichment (PE). The PE scores were motivated by the probabilistic socio-affinity scoring framework of Gavin et al. [11] but also take into account negative evidence (i.e., pairs of proteins where one of them fails to appear as a prey when the other one is used as a bait). These affinity scores encode the reliabilities (confidence) of physical interactions between pairs of proteins. Therefore, the challenge now is to mine meaningful and novel complexes from protein interaction networks derived by combining multiple high-throughput datasets and by making use of these affinity scoring schemes. In this direction, some algorithms have also been proposed [25, 31–34].

In this study, we propose a novel algorithm to derive yeast complexes from weighted (affinity-scored) PPI network and call it ClusterBFS (Cluster with Breadth First Search). ClusterBFS builds clusters in terms of breadth first search algorithm, starting from local seeds and adding nodes that maintain the weighted density of the clusters. The experimental results show that our ClusterBFS method outperforms existing computational methods, such as MCL [31], ClusterONE [32], HC-PIN [33], SPICi [34], and MCODE [14].

## 2. Methods

### 2.1. Preliminaries

Given a weighted network, the goal of our algorithm is to output a set of disjoint dense subgraphs. We model the network as a undirected graph *G* = (*V*, *E*) with a confidence score 0 < *w*_*u*,*v*_ ≤ 1, for every edge (*u*, *v*) ∈ *E*. For any two vertices, *u* and *v* without an edge between them, we set *w*_*u*,*v*_ = 0. For each set of vertices *S* ⊂ *E*, we define its weighted density as the sum of the weights of the edges among them divided by the total number of possible edges (i.e., the density of a set is measure of how close the induced subgraph is to clique, and varies from 0 to 1):

(1) $$\begin{array}{c} D_{w}\left(S\right)=\frac{\sum_{(u,v)\in S}w_{u,v}}{\left|S\right|*\left(\left|S\right|-1\right)/2}. \end{array}$$

### 2.2. Algorithm Overview

**Figure 1:**
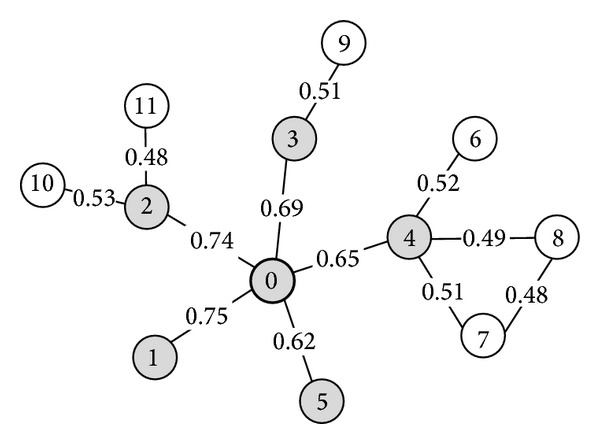
Example to illustrate the clustering process. This example network has 12 vertices, and every edge has confidence. Suppose the weighted density threshold *Td* = 0.2. The vertex 0 is taken as a seed protein and the original cluster 0 is constructed. In the first step of the breadth first search, the vertex 1 has the highest edge weight 0.75 among the neighbors of the vertex 0. We add vertex 1 to the cluster and this cluster {0,1} now has the weighted density 0.75 that is bigger than the density threshold 0.2. Similarly, the vertices 2, 3, 4, and 5 are added to the cluster in sequence and the cluster {0,1, 2,3, 4,5} now has the weighted density 0.23 which is still more than the threshold 0.2. Next, the neighbors of vertex 4 are considered. Of these, vertex 6 has the highest edge weight 0.52 and is added to the cluster. However, the weighted density of the cluster {0,1, 2,3, 4,5, 6} is 0.19 and less than the threshold 0.2. Thus, the vertex 6 is removed and the neighbor of the vertex 3 is examined. Because the weighted value between the vertex 3 and its neighboring vertex 9 is 0.51 and less than 0.52, the vertex 9 is not added to the cluster. When the neighbors of the vertex 2 are checked, the vertex 10 is added to the cluster. Since the weighted density of the cluster {0,1, 2,3, 4,5, 10} is less than 0.2, the vertex 10 is removed. And, likewise, the vertex 11 is not added to the cluster. We stop extending the cluster and output the final cluster {0,1, 2,3, 4,5}. For simplicity, the elimination of redundant clusters is not shown in this figure.

We use a breadth first search approach to build clusters. ClusterBFS builds one cluster at a time, and each cluster is expanded from an original seed protein. The unclustered node is added, if it has the highest edge weight and the density of the cluster remains higher than a user-defined threshold* Td*; otherwise, the cluster is output. The growth process is repeated from different seeds to form multiple, possibly, overlapping groups. Although some overlaps are likely to have biological importance, groups overlapping to a very high extent in comparison to their sizes should likely be discarded. We quantify the extent of overlap between each pair of groups and discard the smaller group, if the overlap score [14] is above a specified threshold. ClusterBFS thus has two parameters:* Td*, the weighted density threshold and *R*. For threshold *R*, we set a firm value 0.8 [32]. (See Figure 1 for a simplified example.)

### 2.3. Seed Selection

Every vertex in the yeast PPI network is used as the seed and is equally important.

### 2.4. Cluster Expansion

After obtaining the seed vertex, we use the breadth first search method to grow each cluster in terms of the weighted density. At each step, we have a current vertex set *C* for the cluster, which initially contains one seed protein *v*. We search for the vertex *u* with maximum value of the edge weight amongst all the unclustered vertices that are adjacent to the seed *v* in breadth first. If the weighted density of the cluster is smaller than a threshold, we stop expanding this cluster and output it. If not, we put vertex *u* into *C* and update the density value. If the density value is smaller than our density threshold* Td*, we do not include *u* in the cluster and output *C*. We repeat this procedure until all vertices in the graph are clustered.  Algorithm 1 illustrates the over framework to detect protein complexes.  Algorithm 2 is the breadth first search procedure.

**Figure 2:**
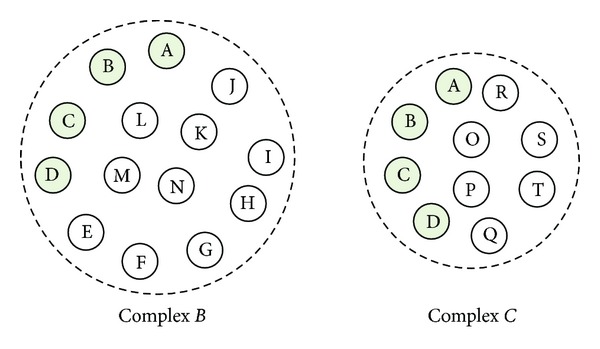
Example to illustrate the Redundancy-filtering. Complex *B* and Complex *C* contain 14 and 10 proteins, respectively. They share 4 proteins A, B, C, and D.

Since all vertices in the graph have been selected as seeds, the clusters produced have large overlaps, which will result in high redundancy. Hence, a Redundancy-filtering procedure is designed to process candidate clusters and finally generate protein complexes by eliminating such kind of redundancy.  Algorithm 3 shows details of the redundancy process. Suppose that SC is the set of all currently detected complexes and *C* = (*V*_*C*_, *E*_*C*_) is a newly identified complex. We will first selected an element *B* = (*V*_*B*_, *E*_*B*_) in SC, which has the highest similarity (OS, overlap score) [14] with *C*. In Algorithm 3, the procedure Redundancy-filtering (*C*) is used to check and decide whether to discard or preserve the newly selected Complex *C*. If *B* and *C* are not quite similar (with OS < *R*), *C* will be inserted into SC in lines 2-3; otherwise, we prefer to preserve the complexes that have larger size in lines 4–8. For instance, suppose Complex *B* of Figure 2 is one complex belonging to the complex set SC and is the most similar to the new complex, that is, Complex *C*. After computing the OS of the two complexes, we obtain a score 0.11 which is less than the threshold *R* = 0.8. So Complex *C* will be inserted into the complex set SC.

## 3. Results

We test the performance of our ClusterBFS method with other five competing algorithms, Markov cluster (MCL) [31], clustering with overlapping neighborhood expansion (ClusterONE) [32], hierarchical clustering on protein interaction network (HC-PIN) [33], speed and performance in clustering (SPICi) [34], and molecular complex detection (MCODE) [14] using the weighted Collins [23] and Krogan datasets [10]. For each algorithm, the final results are obtained after having optimized the algorithm parameters to yield the best possible results. We compare predicted complexes to the reference complex set CYC2008 [35]. We assess the quality of the predicted complexes by two scores: the fraction of protein complexes matched by at least one predicted complex and the maximum matching ratio (MMR) [32]. Our benchmarks show that ClusterBFS outperforms the other approaches on weighted networks, matching more complexes with a higher *F*-measure and providing a better one-to-one mapping with reference complexes in three datasets. To examine the biological relevant of detected complexes we calculate the colocalization and coannotation scores of the entire identified complex set [24]. Comparison of colocalization and coannotation scores of ClusterBFS complexes and other algorithms reveals that ClusterBFS has higher scores on three datasets.

### 3.1. Data Sources

Yeast has long been known as a highly effective model organism for mammalian biological functions and diseases. We evaluate the effectiveness of ClusterBFS using three different yeast PPI weighted networks. The first dataset is prepared by Collins et al. [23]. For the weighted interaction map of Collins et al., we use the top 9074 interactions as suggested by the authors. These interactions among 1622 proteins have very high confidence scores. The second dataset is the Krogan core dataset [10]. It consists of 7123 reliable interactions involving 2708 proteins. We also use Krogan's extended dataset [10] containing 3672 nodes and 14317 edges to test ClusterBFS. For evaluating our identified complexes, the set of real complexes from [35] is selected as benchmark.

### 3.2. Evaluation Measures

One evaluation method we use is to match the generated complexes with known complex set [35] and calculate sensitivity, positive predictive value (PPV), *F*-measure, and MMR, respectively. In information retrieval, positive predictive value is called precision, and sensitivity is called recall. We derive 408 typical complexes including two or more proteins from the CYC2008 [35] as the benchmark complex set and use the same scoring scheme used by [14] to determine how effectively a predicted complex matches a reference complex. If two complexes overlap each other, they must share one or more proteins. The overlap score (OS) of a predicted complex versus a benchmark complex is then a measure of biological significance of the prediction, assuming that the benchmark set of complexes is biologically relevant. The overlap score between a predicted and a real complex is calculated using

(2) $$\begin{array}{c} \text{OS}=\frac{i^{2}}{g\times h}, \end{array}$$

where *i* refers to the number of proteins shared by a predicted complex and a benchmark complex, *g* is the number of proteins in the predicted complex, and *h* is the number of proteins in the benchmark complex. If OS is 1, it means that a complex has the same proteins as a benchmark complex. On the contrary, when OS is more than 0, there is not a shared protein between the predicted complex and the benchmark complex [14].

The number of true positives (TP) is defined as the number of predicted complexes with OS over a threshold value and the number of false positives (FP) is the total number of predicted complexes minus TP. The number of false negatives (FN) equals the number of known complexes not matched by predicted complexes. *Recall* and *Precision* are defined as TP/(TP + FN) and TP/(TP + FP), respectively [14]. *F*-measure, or the harmonic mean of *Recall* and *Precision*, can then be used to evaluate the overall performance of the clustering algorithms:

(3) $$\begin{array}{c} F\text{-measure}=\frac{2\times Recall\times Precision}{Recall+Precision}. \end{array}$$

**Figure 3:**
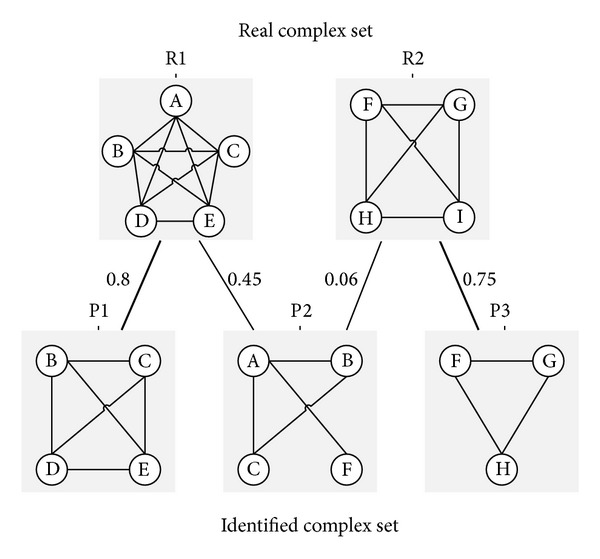
Example to illustrate the maximum matching ratio. R1 and R2 are real complexes, while P1, P2, and P3 are three predictions. An edge connects a reference complex and a predicted complex, if their overlap score is larger than zero. The maximum matching is shown by the thick edges. Note that P2 was not matched to R1 since P1 provides a better match with R1. The maximum matching ratio in this example is (0.8 + 0.75)/2 = 0.775.

MMR score is proposed by Nepusz et al. [32] based on a maximal one-to-one mapping between detected and reference complexes.  Figure 3 illustrates the maximum matching ratio.

Owing to the fact that gold standard protein complex sets are incomplete [36], a predicted complex that does not match any of the reference complexes may belong to a valid but previously uncharacterized complex as well. To this end, the matching measures should be complemented with scores that assess the biological relevance of predicted complexes based on the colocalization and coannotation of the constituent proteins instead of relying on a predefined gold standard. Since protein complexes are formed to perform a specific cellular function, proteins within the same complex tend to share common functions and be colocalized [37]. Generally, higher coannotation and colocalization scores [24] show that proteins within the same protein complexes tend to share higher functional similarity. We employ the software suite* ProCope* (http://www.bio.ifi.lmu.de/Complexes/ProCope/) to compute the colocalization and coannotation scores in our experiment.

### 3.3. Comparison with the Real Complexes on the Collins Dataset

Table 1 shows the number of detected complexes that match at least one real complex over a range of OS thresholds from threshold of 0 to 1.0 (in 0.1 increments). From Table 1, it can be found that the ClusterBFS algorithm detects the most complexes which match at least one known complex over every interval of OS. The second line in Table 1 shows the number of all complexes discovered by each approach. For instance, ClusterBFS predicts altogether 1229 complexes from the Collins dataset, whereas MCL, ClusterONE, HC-PIN, SPICi, and MCODE find 300, 203, 281, 156, and 111 complexes, respectively. The third line displays that when OS is more than 0.1, ClusterBFS curates 829 complexes matched at least one real complex but MCL, ClusterONE, HC-PIN, SPICi, and MCODE merely mine 199, 148, 187, 123, and 98 complexes like that, respectively.  Table 2 gives the number of real complexes which match at least a predicted one.  Table 2 shows that the number of real complexes matched by predicted ones from ClusterBFS is also the largest. The experimental results demonstrate that although ClusterBFS obtains the largest number of complexes, the matched complexes from ClusterBFS are much more than those from the other techniques. That is, ClusterBFS identifies a vast amount of high-quality complexes from the weighted Collins network.

In addition, as shown in Tables 1 and 2, when OS is 1, ClusterBFS identifies 102 real complexes. In other words, 102 predicted complexes from ClusterBFS also belong to the known complex set [35] and are much more than ones from one of the other approaches including MCL, ClusterONE, HC-PIN, SPICi, and MCODE, respectively. More importantly, we observe that the reference set includes 408 real complexes, of which 259 complexes are the small size complex only containing 2 or 3 proteins. Actually, our statistical results (not presented in the tables of the paper) show that, in the 102 real complexes predicted by ClusterBFS, there are 78 small size complexes like that. However, MCL, HC-PIN, SPICi, and MCODE only find 74, 70, 33, and 30 real complexes, respectively. At the same time, MCL, HC-PIN, SPICi, and MCODE just detect 54, 49, 15, and 9 small size real complexes. Since ClusterONE discards the complex candidates that contain less than three proteins, we do not compare it with ClusterBFS. The experimental results show that ClusterBFS has the significant performance advantage over the other algorithms in terms of the identification of small size complexes.

**Figure 4:**
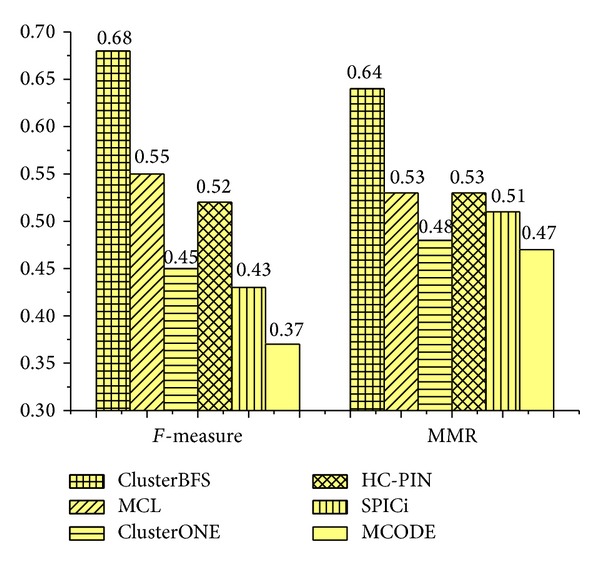
The *F*-measure and MMR of various algorithms on Collins dataset.

Next, we calculate the *F*-measure and MMR scores of the complex sets detected by various techniques. When the *F*-measure is computed, the OS between a predicted complex and a real complex in the benchmark is set as 0.2 [14].  Figure 4 displays the overall comparison according to *F*-measure and MMR. On Collins dataset, the *F*-measure of ClusterBFS is 0.68, which is 23.6%, 51.1%, 30.8%, 58.1%, and 83.8% higher than MCL, ClusterONE, HC-PIN, SPICi, and MCODE, respectively. ClusterBFS can achieve the highest *F*-measure, which shows that our method can predict protein complexes very accurately. From Figure 4, it also can be found that our ClusterBFS method obtains the highest MMR of 0.64, which is 21.8%, 33.3%, 21.8%, 25.5%, and 36.2% higher than MCL, ClusterONE, HC-PIN, SPICi, and MCODE, respectively. That is, ClusterBFS provides a better one-to-one mapping with real complexes in the Collins dataset.

### 3.4. Biological Coherence of Predicted Complexes on Collins Dataset

**Figure 5:**
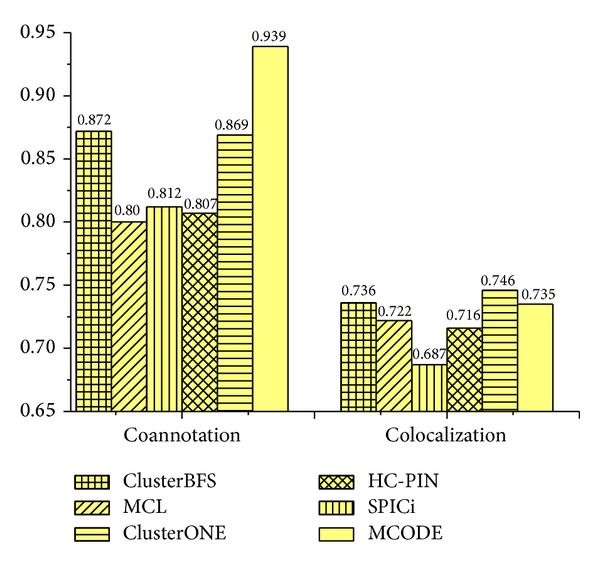
Co-localization and co-annotation scores of complexes identified by various methods on Collins dataset.

Figure 5 shows the colocalization and coannotation scores of complexes detected by various methods. From Figure 5, it can be observed that ClusterBFS has the second highest colocalization score in the five methods after SPICi. However, SPICi cannot handle overlaps. Proteins may have multiple functions, and therefore the corresponding nodes may belong to more than one cluster; for example, 207 of 1,628 proteins in the CYC2008 hand-curated yeast complex dataset [35] participate in more than one complex. So it is important to detect the overlapping complexes. In addition, it can be seen that the coannotation score of ClusterBFS is lower than that of MCODE. It means that the complexes mined by MCODE have the highest biological significance. However, the disadvantage of MCODE is that it only discovers 111 complexes. In other words, the high-quality complexes identified by MCODE are not too many. In terms of the coannotation and colocalization, the complexes predicted by our ClusterBFS method are observed to have comparable quality with those predicted by SPICi and MCODE but much better than those predicted by MCL, ClusterONE, and HC-PIN.

### 3.5. Results Using Krogan Dataset

**Figure 6:**
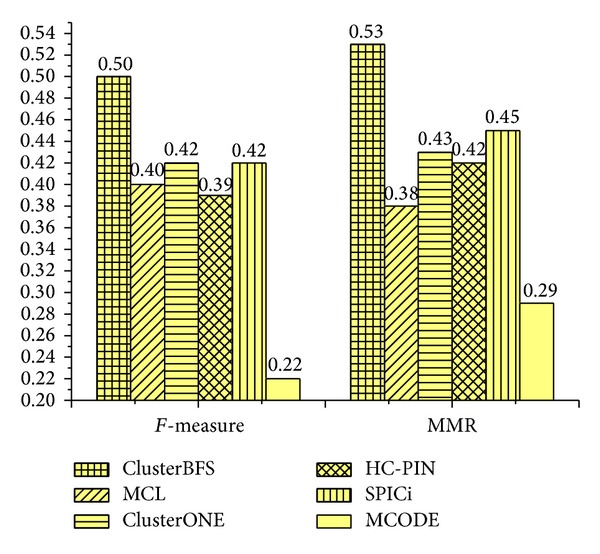
*F*-measure and MMR of various methods for Krogan's core dataset.

**Figure 7:**
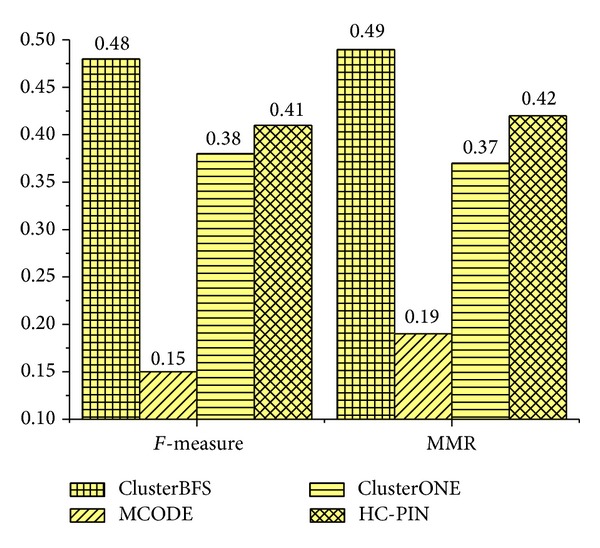
*F*-measure and MMR of various methods for Krogan's extended dataset.

To support the credibility of our method, we perform our ClusterBFS on Krogan's core dataset [10]. The *F*-measure and MMR of each method using this data are shown in Figure 6. The *F*-measure of our ClusterBFS is 0.50, which is 25.0%, 19.0%, 28.2%, 19.0%, and 127.3% higher than MCL, ClusterONE, HC-PIN, SPICi, and MCODE, respectively. From the perspective of MMR, ClusterBFS obtains the score 0.53, which is 39.5%, 23.2%, 26.2%, 17.8%, and 82.76%higher than MCL, ClusterONE, HC-PIN, SPICi, and MCODE, respectively. Additionally, we also test the biological coherence on Krogan's core data and gain the results which are similar to ones from Collins data. For simplicity, the results are not shown in the paper. In order to evaluate whether ClusterBFS can apply to the larger scale dataset, we test it on Krogan's extended dataset. The results are shown in Figure 7. From Figure 7, it can be found that ClusterBFS still obtains the highest scores of *F*-measure and MMR. The experimental results demonstrate that ClusterBFS is not dependent to a particular kind of dataset and can run well on the larger dataset. Besides, it takes around 1 second on an IBM PC with 3.19 GHz processor and 2 GB RAM to generate clusters from Krogan's extended dataset.

### 3.6. Effect of the Parameter* Td*

**Figure 8:**
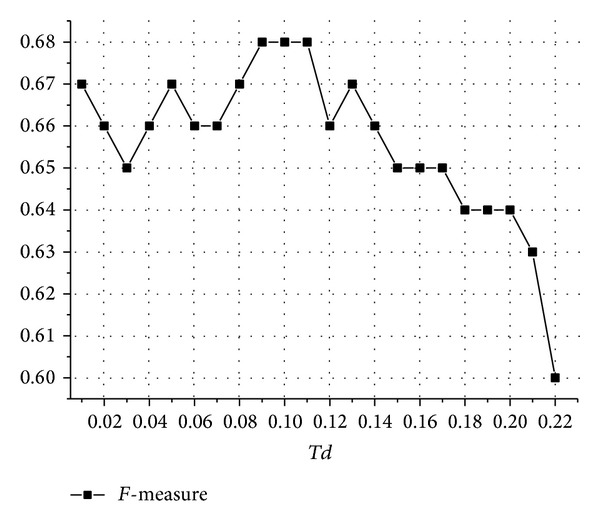
The effect of parameter* Td*.  Figure 8 shows how the variation of parameter* Td* affects the *F*-measure of ClusterBFS.

In this experiment, we study the effect of the thresholds* Td* and *R* on the performance of ClusterBFS. Nepusz et al. have merged pairs of complexes with an overlap score OS larger 0.8 [32]. So we also set the parameter *R* 0.8 in order to investigate the performance of ClusterBFS under different* Td* values.  Figure 8 shows the *F*-measure of ClusterBFS under different values of* Td* based Collins dataset. As shown in Figure 8, when 0.01 ≤* Td* ≤ 0.22, the *F*-measure score of ClusterBFS is more than 0.6 and much higher than those of the other algorithms (See Figure 4). Besides, Figure 8 also shows that when *T* ∈ [0.09,0.11], ClusterBFS gets the highest *F*-measure score. In this interval, the *F*-measure score remains unchanged. Therefore, the parameter* Td* is set as 0.1 in our experiment.

## 4. Conclusion

Protein complexes are important for understanding principles of cellular organization and function. Therefore, much work has been concerned with the prediction of protein complexes from the PPI networks. However, the PPI datasets from high-throughput techniques are flooded with false interactions. In response, some research groups propose a number of data integration and affinity scoring schemes and construct various weighted networks.

In this research, we devise a novel algorithm called ClusterBFS to identify protein complexes from the weighted PPI networks. ClusterBFS derives from the breadth first search method and constitutes protein complexes that originate from a protein seed based on the weighted density. In order to characterize these clusters as protein complexes, we check their biological relevance. This is achieved through some criteria such as *F*-measure, MMR, colocalization, and coannotation measures. The evaluation of our predictions demonstrates the following advantages of ClusterBFS over the compared approaches. First, ClusterBFS has achieved significantly higher *F*-measure and MMR than the existing methods. Thus, our predicted complexes match very well with benchmark complexes. Second, ClusterBFS also performs very well in terms of other measures such as coannotation and colocalization, indicating that ClusterBFS can predict protein complexes very accurately. Last but not least, as mentioned above, the real complex set CYC2008 contains a lot of small complexes and so it is necessary to mine them. In comparison with the other approaches, ClusterBFS discovers much more small size complexes. Our identified complexes, therefore, could be probably the true complexes to help the biologists to get novel biological insights.

## Figures and Tables

**Algorithm 1:**
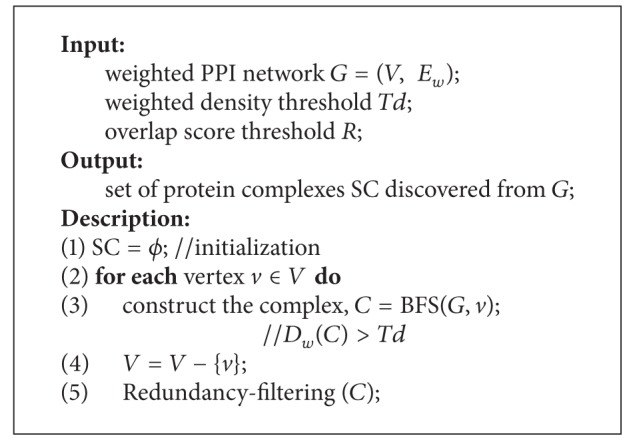
ClusterBFS algorithm.

**Algorithm 2:**
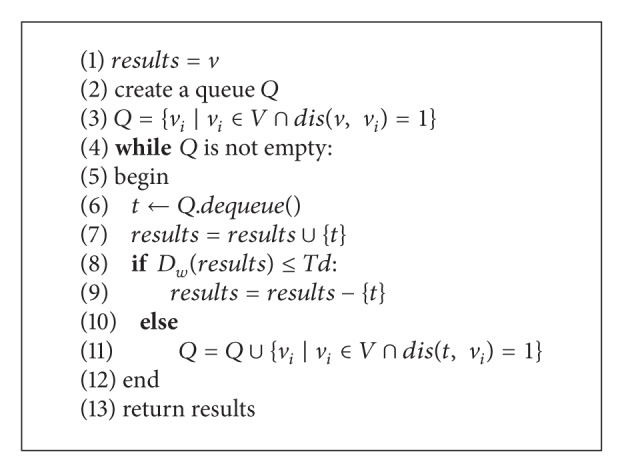
Breadth First Search: BFS(*G*, *v*).

**Algorithm 3:**
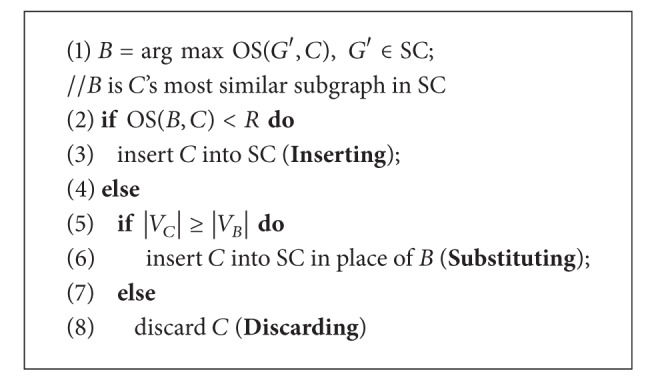
Redundancy-filtering (*C*).

**Table 1:** Comparison of the number of predictions matching at least one known complex.

	ClusterBFS	MCL	ClusterONE	HC-PIN	SPICi	MCODE
OS ≥ 0.0	**1229**	300	203	281	156	111
OS ≥ 0.1	**829**	199	148	187	123	98
OS ≥ 0.2	**697**	182	131	170	114	91
OS ≥ 0.3	**571**	169	123	159	111	88
OS ≥ 0.4	**496**	161	116	150	107	81
OS ≥ 0.5	**398**	146	98	139	95	74
OS ≥ 0.6	**287**	135	87	129	83	64
OS ≥ 0.7	**177**	106	69	97	65	52
OS ≥ 0.8	**128**	93	51	86	51	42
OS ≥ 0.9	**102**	77	38	75	39	34
OS = 1.0	**102**	74	33	70	33	30

**Table 2:** Comparison of the number of real complexes matching at least one detected complex.

	ClusterBFS	MCL	ClusterONE	HC-PIN	SPICi	MCODE
OS ≥ 0.0	**408**	408	408	408	408	408
OS ≥ 0.1	**298**	256	195	244	176	142
OS ≥ 0.2	**269**	227	164	209	142	113
OS ≥ 0.3	**239**	196	149	185	129	103
OS ≥ 0.4	**224**	181	137	171	117	92
OS ≥ 0.5	**206**	159	111	150	104	82
OS ≥ 0.6	**181**	145	96	136	88	70
OS ≥ 0.7	**133**	109	73	100	68	54
OS ≥ 0.8	**115**	94	52	87	53	42
OS ≥ 0.9	**102**	77	38	75	39	34
OS = 1.0	**102**	74	33	70	33	30
